# Exploratory Diagnostic Performance of On-Admission Soluble CD40 Ligand for Distinguishing Acute Pulmonary Embolism from Hospitalization-Requiring Community-Acquired Pneumonia: A Single-Center Observational Study

**DOI:** 10.3390/diagnostics16121877

**Published:** 2026-06-16

**Authors:** Onur Çelik, Adil Furkan Kılıç, Yunus Kuralay, Dursun Erol Afşin

**Affiliations:** 1 Department of Pulmonary Medicine, Erzurum Faculty of Medicine, Health Sciences University, 25240 Erzurum, Türkiye; d.erol@hotmail.com; 2 Department of Internal Medicine, Erzurum Faculty of Medicine, Health Sciences University, 25240 Erzurum, Türkiye; adilfurkanklc@gmail.com; 3 Department of Internal Medicine, Erzurum Regional Training and Research Hospital, 25240 Erzurum, Türkiye; yunuskuralay@gmail.com

**Keywords:** pulmonary embolism, community-acquired pneumonia, soluble CD40 ligand, thrombo-inflammation, platelet activation, exploratory biomarker, comparative biomarker study

## Abstract

**Background/Objectives**: Acute pulmonary embolism (PE) and hospitalization-requiring community-acquired pneumonia (CAP) may present with overlapping clinical, laboratory, and radiological features. Soluble CD40 ligand (sCD40L) is a platelet-derived thrombo-inflammatory mediator that may be influenced by both thrombotic and inflammatory processes. This study retrospectively compared on-admission serum sCD40L concentrations between selected hospitalized patients with established acute PE and selected patients with hospitalization-requiring CAP. **Methods**: This single-center retrospective exploratory comparative biomarker study included 82 hospitalized adults: 48 with computed tomography pulmonary angiography (CTPA)-confirmed acute PE and 34 with hospitalization-requiring CAP defined using CURB-65-supported admission criteria. Stored admission serum samples were used for sCD40L measurement. Between-group comparison was the primary analysis; receiver operating characteristic (ROC) analysis was performed as a secondary exploratory description of the apparent within-sample discriminatory signal. **Results**: sCD40L was higher in acute PE than in hospitalization-requiring CAP (median 821.3 vs. 629.0 pg/mL; *p* < 0.001). ROC analysis demonstrated a strong exploratory within-sample discriminatory signal (AUC 0.951, 95% CI 0.905–0.997). After excluding five patients with recorded antiplatelet or rivaroxaban exposure, the apparent signal remained similar (AUC 0.945; bootstrap 95% CI 0.891–0.984), and sCD40L remained associated with PE in a Firth-penalized model adjusted for platelet count and COPD (OR 3.39 per 50 pg/mL, 95% CI 2.00–7.71; *p* < 0.001). **Conclusions**: In this retrospective selected two-group comparison, on-admission serum sCD40L concentrations were higher in established acute PE than in hospitalization-requiring CAP. ROC-derived estimates should be interpreted only as apparent within-sample discrimination and not as a replacement for D-dimer, clinical probability assessment, or imaging-based PE diagnosis. Prospective validation in unselected suspected-PE cohorts is required before any diagnostic or clinical use can be considered.

## 1. Introduction

Pulmonary embolism (PE) is a major contributor to the global thrombotic disease burden [1], whereas community-acquired pneumonia (CAP) is a common acute respiratory infection that may require hospitalization in patients with greater severity or vulnerability [2]. In emergency and inpatient practice, these conditions may share overlapping clinical features such as dyspnea, chest pain, tachycardia, and hypoxemia. The differential diagnosis between acute PE and hospitalization-requiring CAP at the time of first presentation remains a recurring clinical challenge because clinical findings, radiological abnormalities, inflammatory responses, and D-dimer elevations may overlap [3,4,5]. Timely and accurate diagnosis is critical because the immediate management pathways (anticoagulation versus antimicrobial therapy) differ substantially.

Soluble CD40 ligand (sCD40L) is an 18 kDa member of the tumor necrosis factor (TNF) superfamily released predominantly from activated platelets, with smaller contributions from T lymphocytes and macrophages [6,7,8]. After platelet activation, CD40L is rapidly translocated from intracellular α-granules to the platelet surface and subsequently cleaved into a soluble form that enters the systemic circulation, where it can be quantified in serum or plasma [9,10]. sCD40L exerts both pro-thrombotic and pro-inflammatory effects through its receptor CD40 on endothelial cells, leukocytes, and other platelets, thereby providing a molecular bridge between thrombosis and inflammation [11]. In the contemporary thrombo-inflammation paradigm, sCD40L is increasingly viewed as a key effector of immunothrombosis [12].

Recent mechanistic and clinical studies have linked platelet activation and platelet-derived inflammatory mediators, including sCD40L, to venous thromboembolism and other thrombo-inflammatory conditions [12,13,14,15]. In acute PE, plasma sCD40L has been reported to be higher than in healthy controls [16]. In patients with chronic thromboembolic pulmonary hypertension undergoing pulmonary endarterectomy, elevated preoperative sCD40L levels have been associated with unfavorable postoperative hemodynamics and poor surgical outcomes [17]. In pneumonia and acute respiratory infections, platelet activation and platelet-derived inflammatory mediators have also been documented, particularly in patients with cardiovascular complications [13,14]; however, available studies have not specifically evaluated whether hospitalization-requiring CAP produces an sCD40L profile comparable to that observed in acute PE. This difference in biological context provides a rationale for evaluating whether on-admission sCD40L levels differ between established acute PE and hospitalization-requiring CAP.

Despite this biological plausibility, sCD40L has not been systematically evaluated as a comparative thrombo-inflammatory biomarker between established acute PE and hospitalization-requiring CAP. Importantly, the present study was not designed to test whether sCD40L is superior to D-dimer or to replace standard PE diagnostic algorithms. We hypothesized that on-admission serum sCD40L levels may differ between selected patients with established acute PE and selected patients with hospitalization-requiring CAP. The primary objective was to compare sCD40L concentrations between these two hospitalized cohorts. Secondary exploratory objectives were to describe the apparent within-sample discriminatory signal of sCD40L and to assess whether the observed signal persisted after exclusion of patients with documented antithrombotic exposure and after adjustment for platelet count and COPD. Accordingly, any diagnostic-performance estimates in this manuscript should be understood as exploratory apparent within-sample discrimination in a selected two-gate comparison, not as validation of a clinically actionable diagnostic test.

## 2. Materials and Methods

### 2.1. Reporting Guidelines and Ethical Approval

This observational exploratory comparative biomarker study was reported primarily in accordance with the Strengthening the Reporting of Observational Studies in Epidemiology (STROBE) Statement [18]. Because secondary exploratory ROC-based analyses were also performed, relevant items from the Standards for Reporting of Diagnostic Accuracy Studies (STARD) 2015 checklist [19] were additionally addressed. Completed STROBE and STARD 2015checklists are provided as Supplementary Materials. The study was approved by the Health Sciences University, Erzurum Faculty of Medicine, Scientific Research Ethics Committee (approval code: 2023/08-95; approval date: 13 December 2023) and was conducted in accordance with the Declaration of Helsinki.Because of the retrospective design, the requirement for written informed consent was waived by the ethics committee. All patient data were de-identified prior to analysis.

### 2.2. Study Design and Setting

We conducted a single-center retrospective exploratory comparative biomarker study using existing clinical, laboratory, imaging, and stored-serum data from Erzurum Training and Research Hospital, a tertiary care institution serving a regional referral population in eastern Türkiye. Medical records of adult inpatients (≥18 years) admitted to the Chest Diseases Clinic between December 2023 and December 2024 were retrospectively reviewed for eligibility. Eligibility criteria and the analytic plan were defined before statistical analysis of the assembled retrospective dataset. During the study period, 50 hospitalized patients with acute PE and 144 hospitalized patients with CAP were identified in the Chest Diseases Clinic. Because CAP admissions substantially outnumbered PE admissions, all available PE cases and a chronological feasibility sample of the first 40 CAP admissions meeting the clinical screening criteria were reviewed to obtain a retrospective exploratory comparator group of similar order of magnitude to the PE cohort. This comparator sampling strategy was used for feasibility and was not intended to represent the full spectrum of hospitalized CAP. After applying the eligibility criteria, 2 PE patients were excluded because of urinary tract infection (*n* = 1) or previous arterial thrombosis (*n* = 1), and 6 CAP patients were excluded because of severe valvular heart disease (*n* = 2), recent cerebrovascular accident within 6 months (*n* = 1), previous arterial thrombosis (*n* = 1), or extrapulmonary infection (*n* = 2). The final analytic cohort consisted of 82 patients (48 PE and 34 CAP). The study flow and sensitivity cohort derivation are summarized in Supplementary Figure S1. Although ROC analysis was reported because the study explored apparent discrimination between two established disease groups, this design does not represent an unselected suspected-PE diagnostic accuracy study. Routinely obtained stored admission serum samples suitable for sCD40L measurement were available for all patients who met the final eligibility criteria; therefore, final inclusion did not depend on selective serum-sample availability.

### 2.3. Case Definitions

#### 2.3.1. Diagnostic Criteria for Acute Pulmonary Embolism

Acute pulmonary embolism was diagnosed based on the presence of at least one intraluminal filling defect in the main, lobar, segmental, or subsegmental pulmonary arteries on multidetector CTPA performed using a 64-slice multidetector CT scanner (Aquilion 64, Toshiba Medical Systems, Otawara, Japan) and a standardized protocol (slice thickness ≤ 1.25 mm, contrast bolus 80–100 mL of iodinated contrast, automated bolus tracking with region of interest in the main pulmonary artery). All CTPA examinations were independently interpreted by board-certified thoracic radiologists with at least five years of experience. Symptom onset within 14 days of admission was required to define the episode as acute. Patients with chronic thromboembolic pulmonary hypertension (CTEPH), recurrent PE on stable anticoagulation, or radiologically confirmed prior PE without an acute event were not eligible.

#### 2.3.2. Diagnostic Criteria for Hospitalization-Requiring Community-Acquired Pneumonia

CAP was defined by compatible clinical features and radiological pulmonary infiltrates present at admission. In this study, hospitalization-requiring CAP was operationally defined as CAP requiring inpatient management according to CURB-65-supported admission criteria. CURB-65 was retrospectively derived from admission data using five variables: new confusion, blood urea nitrogen > 19 mg/dL (>7 mmol/L urea), respiratory rate ≥ 30/min, systolic blood pressure < 90 mmHg or diastolic blood pressure ≤ 60 mmHg, and age ≥ 65 years. A CURB-65 score ≥ 2 was considered supportive of hospital-based care, consistent with guideline-supported use of CURB-65 together with clinical judgment to inform place-of-care decisions. Patients with lower CURB-65 scores were eligible only when inpatient care was clinically required because of hypoxemia, comorbidity burden, extensive radiological involvement, or failure of outpatient management. Patients with hospital-acquired or ventilator-associated pneumonia, viral pneumonia, immunosuppression, or active malignancy under treatment were not eligible [2,20,21].

### 2.4. Handling of Overlapping Conditions

Patients with concomitant clinical and radiological evidence of both acute PE and CAP (“infarct pneumonia”) would have introduced overlapping platelet activation and inflammatory pathways, making it impossible to attribute observed sCD40L differences to either thrombosis or infection alone. For the present retrospective analysis, the comparison was defined before statistical testing to evaluate sCD40L levels between two clinically and pathophysiologically distinct hospitalized conditions; therefore, patients with concomitant PE and CAP were excluded by design. During the study period, no otherwise-eligible patient with concurrent clinical and radiological evidence of both conditions was identified.

### 2.5. Imaging Strategy in the Hospitalization-Requiring CAP Group

In the hospitalization-requiring CAP group, CTPA was performed in 29 of 34 patients (85.3%) as part of the diagnostic work-up to exclude concomitant acute PE. All CTPA examinations were independently reviewed, and PE was excluded based on the absence of intraluminal filling defects in the pulmonary arterial tree. In the remaining 5 hospitalization-requiring CAP patients, CTPA could not be performed because of acute kidney injury that contraindicated iodinated contrast administration. Ventilation-perfusion (V/Q) scintigraphy was not performed in these patients for two reasons: (1) the V/Q scintigraphy service at our institution operated only on weekdays during regular working hours, and three of these five patients presented during evening or weekend periods when V/Q was unavailable; (2) in the remaining two patients, the treating clinical team judged the clinical pre-test probability of PE to be low based on absent typical PE features (no leg pain or swelling, absence of unexplained tachycardia or hypoxemia, low revised Geneva score). In these 5 patients, lower-extremity venous Doppler ultrasonography demonstrated no evidence of deep vein thrombosis at the index admission. A subsequent one-year follow-up of the institutional electronic medical records—including all outpatient clinic visits, emergency department re-admissions, and inpatient stays at our institution—identified no diagnosis of pulmonary embolism, deep vein thrombosis, or related thrombotic event. All 5 patients also demonstrated appropriate clinical response to antimicrobial therapy alone without anticoagulation, further supporting an infectious rather than thrombotic etiology.

For descriptive assessment of anatomical disease extent, additional imaging-based variables were recorded. For exploratory assessment of PE extent, the pulmonary artery obstruction index (PAOI), also known as the Qanadli CT obstruction index, was retrospectively assessed on admission CTPA images/reports. The pulmonary arterial tree was considered to comprise 10 segmental arteries in each lung, yielding 20 segmental arteries in total. For thrombi located in proximal pulmonary arteries, including the main or lobar pulmonary arteries, the score corresponded to the number of segmental arterial branches distal to the thrombus. Isolated segmental thrombi were assigned 1 point each. The degree of obstruction was scored as 1 for partial obstruction and 2 for complete obstruction. The maximum possible raw score was 40, and PAOI was expressed as a percentage using the formula: PAOI (%) = total obstruction score/40 × 100. For exploratory descriptive analyses, PAOI was categorized into three obstruction-burden groups: low (<20%), intermediate (20–37.5%), and high (≥40%), consistent with previously published Qanadli obstruction index categories [22]. For the CAP cohort, admission imaging reports were reviewed to classify radiological pneumonia extent. Limited CAP was defined as unilateral single-lobar involvement, whereas extensive CAP was defined as multilobar and/or bilateral involvement.

### 2.6. Comorbidity Definitions and Exclusion Criteria

To reduce biological confounding in this exploratory biomarker study, patients with conditions known or expected to substantially alter platelet activation, thrombo-inflammatory activity, or circulating sCD40L concentrations were not eligible: acute coronary syndrome (per the Fourth Universal Definition of Myocardial Infarction [23]), clinically documented congestive heart failure or decompensated heart failure (per the 2021 ESC Guidelines for Heart Failure [24]), significant valvular heart disease (severe stenosis or regurgitation documented on echocardiography, per ACC/AHA 2020 [25]), symptomatic or imaging-confirmed peripheral arterial disease, cerebrovascular disease within the preceding six months, extrapulmonary infection, prior venous thromboembolism in the CAP group, or prior PE without acute presentation. Non-exclusionary comorbidities recorded for descriptive purposes included chronic kidney disease (estimated glomerular filtration rate <60 mL/min/1.73 m^2^ by the 2021 CKD-EPI equation [26]), malignancy, COPD, diabetes mellitus, and arterial hypertension. Comorbidity exclusions were ascertained from documented diagnoses in existing medical records. Because platelet-modifying therapies may influence circulating sCD40L concentrations, available medication fields were retrospectively reviewed for documented antiplatelet therapy and rivaroxaban use at admission. These medication exposures were described between groups and were addressed through a medication-exclusion sensitivity analysis.

### 2.7. Pre-Test Probability Assessment and PE Severity Stratification

Clinical pre-test probability was retrospectively calculated from admission records using the revised Geneva score [27]. PE severity was retrospectively stratified using the Pulmonary Embolism Severity Index (PESI) [28] and documented hemodynamic status. Based on PESI class together with hemodynamic status, patients were further categorized as low-, intermediate-, or high-risk PE, consistent with contemporary PE risk stratification principles [29].

### 2.8. Echocardiographic Assessment

According to available clinical records, all PE patients had undergone transthoracic echocardiography (TTE) within 24 h of admission for evaluation of right ventricular dysfunction (RVD). RVD was defined as the presence of at least one of: tricuspid annular plane systolic excursion (TAPSE) < 17 mm, right-to-left ventricular end-diastolic diameter ratio ≥ 0.9, or quantitative evidence of right ventricular dilatation or hypokinesia [30,31]. In the hospitalization-requiring CAP group, TTE was performed selectively when clinically indicated rather than as part of a standardized study protocol; therefore, no between-group RV comparison was attempted, and the RVD analysis was defined as a within-PE-group exploratory analysis before statistical testing of the assembled retrospective dataset.

### 2.9. Blood Sampling and Biomarker Measurement

Routinely obtained stored admission venous serum samples were available for all patients included in the analytic cohort. According to available medical records, samples were obtained at or near the time of hospital admission. When timing was documented, sampling appeared to precede therapeutic anticoagulation or systemic antibiotic therapy that could influence sCD40L kinetics; however, because treatment and sampling times were reconstructed retrospectively from medical records, undocumented timing variability cannot be excluded. Serum samples were processed and stored at −80 °C according to standard laboratory procedures prior to batch analysis. Because sample handling was reconstructed retrospectively from laboratory records, exact pre-analytical processing times could not be verified for every sample. sCD40L was not part of routine clinical testing and was measured specifically for this research study using stored admission serum samples. Serum sCD40L concentrations were measured using a commercial sandwich enzyme-linked immunosorbent assay (ELISA) kit (SunLong Biotech Co., Ltd., Hangzhou, China; catalog no. SL1599Hu), according to the manufacturer’s instructions. The manufacturer-stated assay range was 10–800 pg/mL for the diluted samples, corresponding to an undiluted serum-equivalent range of 50–4000 pg/mL after application of the protocol-specified 1:5 dilution factor used throughout this manuscript. The manufacturer-reported sensitivity was 2.5 pg/mL, with an intra-assay coefficient of variation (CV) of <10% and an inter-assay CV of <12%. All ELISA measurements were performed in duplicate. Because the manufacturer protocol requires a fixed 1:5 sample dilution, we report back-calculated undiluted serum-equivalent concentrations throughout the manuscript (measured value × 5). Discrimination metrics and classification counts are unchanged by this linear scaling. D-dimer, fibrinogen, procalcitonin, C-reactive protein, troponin I, complete blood count parameters, serum albumin, and lactate dehydrogenase were measured using standard clinical laboratory methods on the same admission sample.

### 2.10. Statistical Analyses

Routine statistical analyses were performed using SPSS version 31.0.1.0 (IBM Corp., Armonk, NY, USA). The Firth-penalized logistic regression analysis was performed in R version 4.5.2 (R Foundation for Statistical Computing, Vienna, Austria) using the logistf package. This method was used for the clinically motivated medication-exclusion sensitivity analysis because of the modest cohort size and sparse recorded antithrombotic exposure. Distributional assumptions were assessed using visual inspection of histograms and Q–Q plots together with normality testing, as appropriate. Continuous variables are presented as mean ± standard deviation (SD) or median (interquartile range, IQR) according to data distribution, and categorical variables as counts and percentages. Between-group comparisons were performed using the independent-samples *t* test or Welch’s *t* test for normally distributed continuous variables, the Mann–Whitney U test for non-normally distributed continuous variables, and the chi-square or Fisher’s exact test for categorical variables. Exploratory extent-stratified analyses compared sCD40L levels across PE obstruction-burden categories using the Kruskal–Wallis test and between CAP radiological extent groups using the Mann–Whitney U test. A sensitivity comparison of the full PE cohort versus the extensive CAP subgroup was also performed using the Mann–Whitney U test. Spearman correlation analysis was used to assess the relationship between sCD40L and D-dimer in the overall cohort and within each diagnostic group.

Receiver operating characteristic (ROC) curve analysis was performed only as a secondary exploratory analysis to describe the apparent within-sample discriminatory signal of sCD40L in this selected cohort. Area under the curve (AUC) values are reported with 95% confidence intervals (CIs), and the optimal cut-off in the full cohort was determined using the Youden index for descriptive purposes only. The cut-off was not intended to be clinically actionable or to replace D-dimer, clinical probability assessment, or imaging-based diagnostic algorithms. In the medication-exclusion sensitivity cohort, the AUC was re-estimated after excluding patients with documented antiplatelet therapy or rivaroxaban use at admission; a percentile bootstrap 95% CI was obtained using 10,000 stratified resamples. No formal a priori sample-size calculation was performed because this was a retrospective exploratory study; all patients in the screened cohort who met the retrospectively defined eligibility criteria before analysis had routinely obtained stored admission serum available and were included in the analytic cohort.

To directly examine the potential influence of recorded antithrombotic exposure, the availability of documented antiplatelet therapy and rivaroxaban use at admission was first compared between diagnostic groups. Because only five patients had a recorded exposure, precise covariate estimation for medication use was not feasible. A medication-exclusion sensitivity cohort was therefore formed by excluding these five patients, and Firth-penalized logistic regression was performed with acute PE as the dependent variable (PE = 1; hospitalization-requiring CAP = 0). The parsimonious model included sCD40L per 50 pg/mL increase, platelet count per 50 × 10^3^/µL increase, and COPD status because COPD was the principal measured baseline imbalance between groups. Odds ratios (ORs) are reported with profile-likelihood 95% CIs. Performance of the original exploratory threshold in this sensitivity cohort is detailed in Supplementary Table S2.

## 3. Results

### 3.1. Baseline Characteristics

The final analytic cohort consisted of 82 patients: 48 (58.5%) with acute PE and 34 (41.5%) with hospitalization-requiring CAP. Baseline demographic, clinical, and comorbidity characteristics are summarized in Table 1. Sex distribution and mean age were similar between groups. COPD was more frequent in the hospitalization-requiring CAP group than in the PE group (50.0% vs. 20.8%; Fisher’s exact *p* = 0.008). Recorded antithrombotic exposure at admission was present in 1/48 PE patients and 4/34 CAP patients (*p* = 0.155), and was addressed in the medication-exclusion sensitivity analysis.

### 3.2. PE Severity Stratification

Within the PE cohort, the revised Geneva score classified 2 patients (4.2%) as low probability, 23 (47.9%) as intermediate, and 23 (47.9%) as high probability of PE; the median Geneva score was 10 (IQR 7–12). PESI class distribution was: class I in 10 (20.8%), class II in 3 (6.2%), class III in 2 (4.2%), class IV in 13 (27.1%), and class V in 20 (41.7%). Based on PESI class and hemodynamic status, 11 patients (22.9%) were categorized as high-risk PE, 31 (64.6%) as intermediate-risk, and 6 (12.5%) as low-risk (Table 2).

### 3.3. Exploratory Disease-Extent and D-Dimer Correlation Analyses

Disease extent was descriptively stratified in both cohorts (Supplementary Table S3). In the PE cohort, PAOI/CT obstruction-burden data were available for all 48 patients. Low, intermediate, and high obstruction burden were observed in 19, 10, and 19 patients, respectively. Median serum-equivalent sCD40L levels were 828.5 pg/mL (Q1–Q3: 772.5–927.8) in the low obstruction group, 807.8 pg/mL (Q1–Q3: 773.5–915.4) in the intermediate obstruction group, and 821.5 pg/mL (Q1–Q3: 777.0–937.5) in the high obstruction group, with no significant difference across categories (Kruskal–Wallis *p* = 0.984). In the CAP cohort, radiological pneumonia extent was available for all 34 patients. Limited CAP was present in 15 patients, whereas extensive CAP, defined as multilobar and/or bilateral involvement, was present in 19 patients. Median serum-equivalent sCD40L levels were 548.5 pg/mL (Q1–Q3: 495.3–660.5) in limited CAP and 640.0 pg/mL (Q1–Q3: 622.0–721.5) in extensive CAP (Mann–Whitney U *p* = 0.012). As an exploratory sensitivity comparison addressing pneumonia extent, sCD40L levels in the full PE cohort were compared with those in the extensive CAP subgroup. Median serum-equivalent sCD40L was 821.3 pg/mL (Q1–Q3: 770.8–936.0) in PE and 640.0 pg/mL (Q1–Q3: 622.0–721.5) in extensive CAP (Mann–Whitney U = 848.5, *p* < 0.001). In addition, sCD40L did not significantly correlate with D-dimer in the overall cohort (Spearman rho = −0.168, *p* = 0.144), PE cohort (rho = −0.248, *p* = 0.109), or CAP cohort (rho = 0.001, *p* = 0.996) (Supplementary Table S4). Because these analyses were exploratory and underpowered, they should be interpreted descriptively.

### 3.4. Laboratory Comparison

Most continuous variables showed non-normal distributions; therefore, between-group comparisons used the Mann–Whitney U test. sCD40L was significantly higher in the PE group than in the hospitalization-requiring CAP group (median 821.3 vs. 629.0 pg/mL, serum-equivalent values; Mann–Whitney U = 79.5, *p* < 0.001). Neutrophil percentage was significantly higher in the hospitalization-requiring CAP group (median 77.0% vs. 61.0%; *p* = 0.002). A small between-group difference was observed for MCHC (*p* = 0.047). Serum albumin was significantly higher in the PE group (median 39.5 vs. 34.5 g/L; *p* = 0.005), consistent with the more pronounced acute-phase response in hospitalization-requiring pneumonia. In contrast, D-dimer, fibrinogen, MCV, P-LCR, lymphocyte percentage, troponin I, CRP, procalcitonin, WBC, platelet count, MPV, PDW, NLR, PLR, SII, and LDH did not differ significantly between groups (all *p* > 0.05). A full comparison of laboratory parameters is provided in Table 3.

### 3.5. Secondary Exploratory ROC Analysis of sCD40L

**Figure 1:**
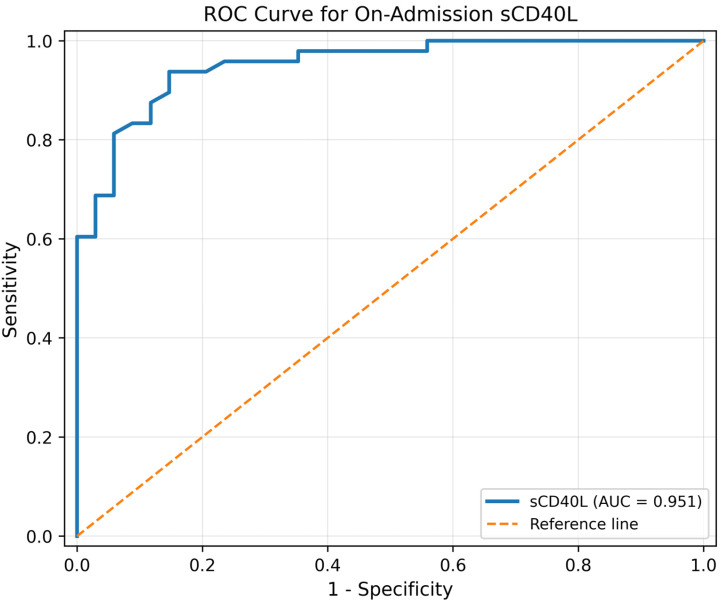
Receiver operating characteristic (ROC) curve from the secondary exploratory within-sample analysis of on-admission serum soluble CD40 ligand (sCD40L) in the selected PE-versus-CAP cohort. The diagonal line represents no discrimination (AUC = 0.50). The observed AUC was 0.951 (95% CI 0.905–0.997).

**Figure 2:**
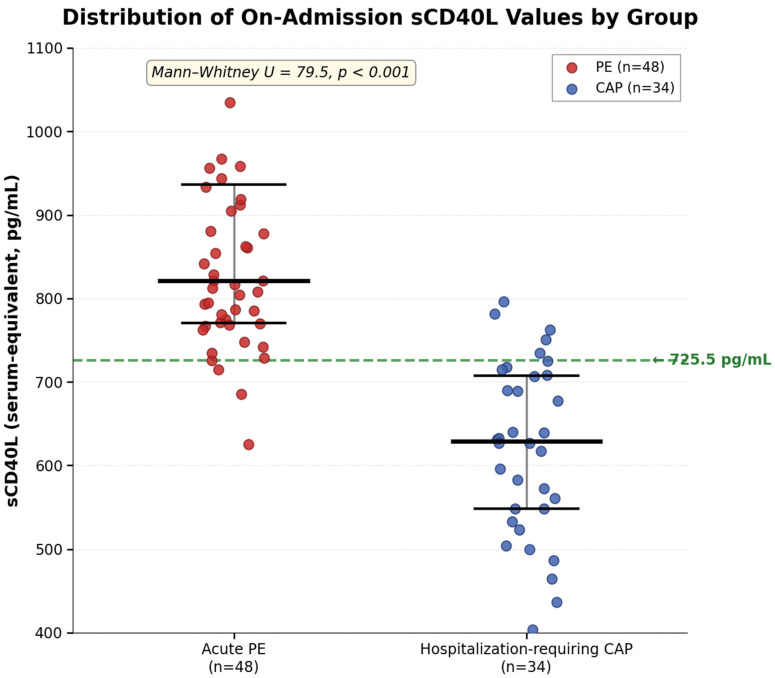
Distribution of on-admission serum soluble CD40 ligand (sCD40L) values in patients with acute pulmonary embolism (PE, *n* = 48) and hospitalization-requiring community-acquired pneumonia (CAP, *n* = 34). Dots represent individual patients; horizontal bars indicate medians and interquartile ranges. The dashed line indicates the Youden-derived exploratory threshold of 725.5 pg/mL. Values are reported as serum-equivalent concentrations. The between-group comparison shown in the figure corresponds to Mann–Whitney U = 79.5, *p* < 0.001.

As a secondary exploratory analysis, ROC analysis was used to describe the apparent within-sample discriminatory signal of sCD40L between the two established disease groups. ROC analysis demonstrated a promising exploratory discriminatory signal of sCD40L for distinguishing acute PE from hospitalization-requiring CAP in the full cohort (AUC 0.951, 95% CI 0.905–0.997; Gini index 0.903). The Youden-derived cut-off was 725.5 pg/mL (Figure 1). The individual distribution of sCD40L values is presented in Figure 2, showing an overall between-group shift with a remaining zone of overlap. Exploratory within-sample classification at this cut-off is shown in Table 4. At 725.5 pg/mL, sensitivity was 93.8% (45/48; 95% CI 83.2–97.9) and specificity was 85.3% (29/34; 95% CI 69.9–93.6), with positive likelihood ratio 6.38 (95% CI 2.83–14.37) and negative likelihood ratio 0.07 (95% CI 0.02–0.22). The apparent positive predictive value was 90.0% (45/50; 95% CI 78.6–95.7) and the apparent negative predictive value was 90.6% (29/32; 95% CI 75.8–96.8). Because PPV and NPV depend on disease prevalence and this study used a selected two-gate case–control design rather than an unselected diagnostic cohort, PPV and NPV should be interpreted only as within-sample descriptive measures and should not be extrapolated directly to clinical practice. The threshold should be regarded as exploratory and assay-specific rather than clinically actionable; additional exploratory thresholds are provided in Supplementary Table S1. Because the cohort was selected and two-gate in design, these estimates should not be interpreted as real-world diagnostic performance in unselected patients with suspected PE.

### 3.6. Medication-Exclusion Sensitivity Analysis and Firth-Penalized Modeling

The available medication variables identified documented antithrombotic exposure at admission in five patients: antiplatelet therapy in one PE patient and rivaroxaban therapy in four hospitalization-requiring CAP patients (Table 5). After excluding these five patients, the sensitivity cohort consisted of 77 patients (47 PE and 30 CAP). In this cohort, the apparent within-sample discriminatory signal of sCD40L remained similar to that in the full cohort (AUC 0.945; bootstrap 95% CI 0.891–0.984). At the original exploratory cut-off of 725.5 pg/mL, sensitivity was 93.6% (44/47) and specificity was 83.3% (25/30); additional classification details for this sensitivity cohort are provided in Supplementary Table S2.

The Firth-penalized logistic regression results are shown in Table 6.

In the medication-exclusion Firth-penalized model, sCD40L remained associated with acute PE after adjustment for platelet count and COPD (OR 3.39 per 50 pg/mL increase, 95% CI 2.00–7.71; *p* < 0.001). Platelet count and COPD were not statistically associated with PE in this sensitivity cohort. Because only five patients had recorded antithrombotic exposure, the medication-exclusion analysis is more interpretable than fitting a medication coefficient with an extremely imprecise estimate.

### 3.7. Exploratory Within-Sample ROC Summaries of Selected Biomarkers

Exploratory within-sample ROC summaries of selected biomarkers at their Youden-derived thresholds are presented in Table 7; for neutrophil percentage, the direction of discrimination was reversed, with higher values favoring hospitalization-requiring CAP rather than PE.

### 3.8. Exploratory RVD Analysis Within the PE Cohort

Among PE patients, none of the evaluated laboratory parameters differed significantly between those with and without right ventricular dysfunction (all *p* > 0.05; Table 8). This subgroup analysis was exploratory and should be interpreted cautiously.

## 4. Discussion

In this single-center retrospective exploratory comparative biomarker study of 82 hospitalized patients, on-admission serum sCD40L levels were higher in established acute PE than in hospitalization-requiring CAP. ROC analysis showed a strong within-sample discriminatory signal (AUC 0.951), and the signal remained similar after exclusion of patients with recorded antithrombotic exposure. However, these findings should be interpreted as exploratory evidence of a between-group thrombo-inflammatory biomarker difference rather than as proof of clinical diagnostic utility. The selected two-gate design, modest sample size, absence of external validation, and incomplete radiological PE exclusion in a small CAP subset preclude direct translation into routine suspected-PE diagnostic pathways. Thus, any diagnostic-performance terminology in the present manuscript refers only to apparent exploratory discrimination within this selected PE-versus-CAP cohort, not to validated diagnostic accuracy in routine suspected-PE pathways.

### 4.1. Biological Rationale Within the Thrombo-Inflammation Paradigm

Our findings can be interpreted within the contemporary framework of thrombo-inflammation and immunothrombosis [12,32]. Experimental and clinical studies support the involvement of CD40L/CD40 signaling and platelet activation in inflammation, thrombo-inflammation, and thrombotic disease contexts [33], while recent evidence also supports an important role of procoagulant platelet activation in venous thrombosis and pulmonary embolism [34]. sCD40L is predominantly platelet-derived [6,7,8,9,10,11]. Consistent with this thrombo-inflammatory framework, Kaya et al. (2012) reported nearly 4-fold higher plasma sCD40L levels in acute PE patients than in healthy controls [16], and Shigeta et al. (2021) showed that preoperative sCD40L predicts outcomes in CTEPH patients undergoing pulmonary endarterectomy [17]. However, sCD40L is not thrombosis-specific, and inflammatory conditions such as pneumonia may also influence platelet activation and circulating sCD40L. In CAP, platelet activation has been associated with cardiovascular complications [13], whereas in SARS-CoV-2 infection, platelet-derived sCD40L elevation has been reported as part of an inflammatory platelet signature [14]. Recent experimental and clinical evidence from SARS-CoV-2 infection further supports the role of sCD40L-mediated platelet activation and thrombo-inflammation in this specific setting [35]. sCD40L has also been investigated as a biomarker in acute coronary syndromes [36]. Within this framework, the higher sCD40L concentrations observed in our acute PE group than in hospitalization-requiring CAP do not necessarily contradict the inflammatory biology of sCD40L. Rather, they suggest that, in this selected cohort, established acute PE may have been associated with a stronger circulating platelet-derived thrombo-inflammatory signal than hospitalization-requiring CAP. The observed differences may reflect differences in platelet activation, thrombotic burden, timing of sampling, or other unmeasured thrombo-inflammatory pathways, and should not be interpreted as definitive evidence that sCD40L reflects thrombosis rather than inflammation.

Because anatomical disease extent may influence fibrin turnover and thrombo-inflammatory biomarker concentrations, we performed descriptive stratification of both PE obstruction burden and CAP radiological extent. sCD40L did not differ across PE obstruction-burden categories, suggesting that the between-group signal was not simply explained by CT obstruction burden within the PE cohort. In contrast, sCD40L was higher in extensive than limited CAP, supporting the concept that inflammatory disease extent may influence sCD40L concentrations. Importantly, sCD40L remained higher in the full PE cohort than in the extensive CAP subgroup, and sCD40L did not significantly correlate with D-dimer in the overall cohort or within either diagnostic group. These exploratory findings suggest that sCD40L may capture biological information not fully overlapping with fibrin degradation; however, they should not be interpreted as definitive evidence of disease specificity. Future prospective studies should include systematic quantification of thrombus burden and pneumonia volume to determine whether sCD40L reflects disease extent, disease type, or overall thrombo-inflammatory activity.

### 4.2. Recorded Antithrombotic Exposure and Between-Group Comorbidity Imbalance

Medication-related confounding is particularly relevant because sCD40L is platelet-derived. In the available records, documented antithrombotic exposure at admission was uncommon but not absent: one PE patient had recorded antiplatelet therapy and four CAP patients had recorded rivaroxaban use. Excluding these five participants resulted in an AUC of 0.945 and preserved the association between sCD40L and PE in Firth-penalized analysis adjusted for platelet count and COPD. COPD was more common in CAP than PE in the final dataset (50.0% vs. 20.8%); its inclusion in the sensitivity model did not materially attenuate the sCD40L estimate. These analyses address measured medication exposure and the principal measured comorbidity imbalance, but cannot eliminate residual confounding from medications or cardiovascular conditions not comprehensively captured in the retrospective dataset. Diabetes was recorded only in the CAP group; if diabetes increases platelet activation, this imbalance would be expected to attenuate rather than exaggerate the observed higher sCD40L levels in PE, although residual confounding cannot be excluded.

### 4.3. Specificity of the sCD40L Signal Beyond Routine Platelet and Inflammatory Parameters

Platelet count, MPV, and PDW did not significantly differ between groups in the available dataset. This suggests that the observed sCD40L difference is not simply mirrored by these routine platelet indices. Nevertheless, because functional platelet activity is not fully characterized by standard indices and medication exposure was incompletely characterized beyond recorded antiplatelet therapy and rivaroxaban, mechanistic interpretation should remain cautious.

### 4.4. Clinical Implications

In contemporary practice, suspected PE diagnosis relies on sequential diagnostic strategies integrating clinical probability assessment, D-dimer testing in appropriate pre-test probability groups, and confirmatory imaging, most commonly CTPA [29,37,38]. Recent reviews continue to emphasize probability-adapted D-dimer strategies as tools for safely excluding PE in selected patients rather than as stand-alone confirmatory tests [37,38]. Therefore, the present findings should not be interpreted as supporting replacement of D-dimer or imaging-based diagnosis by sCD40L. Rather, they identify sCD40L as a candidate thrombo-inflammatory biomarker that may warrant prospective evaluation in larger, unselected emergency department cohorts, particularly in patients with overlapping respiratory presentations in whom PE and pneumonia are both considered [5,39]. The ROC-derived cut-off of 725.5 pg/mL was selected for maximal discrimination within this dataset and should be considered assay-specific and exploratory until externally validated.

From a practical and translational perspective, sCD40L testing cannot currently be positioned within routine acute PE diagnostic algorithms. Contemporary PE pathways rely on clinical probability assessment, D-dimer testing in appropriate pre-test probability groups, and confirmatory imaging, most commonly CTPA [29,37,38]. In contrast, sCD40L was measured in the present study by ELISA in stored serum samples using batch analysis, which is fundamentally different from the rapid turnaround required for emergency PE decision-making. In addition, sCD40L concentrations may be influenced by sample matrix, pre-analytical handling, processing time, and ELISA methodology, limiting direct comparability across laboratories [40,41]. Therefore, the present findings do not support replacing D-dimer, clinical probability assessment, or CTPA with sCD40L. If validated prospectively and translated into a standardized rapid assay platform, sCD40L could be explored as an adjunctive thrombo-inflammatory biomarker in selected patients with overlapping PE and pneumonia features; until then, it should remain a research biomarker rather than a clinically actionable test.

### 4.5. Comparison with the Related Thrombosis Biomarker Literature

Our findings extend prior observations of elevated sCD40L in acute PE [16] and CTEPH [17] by providing preliminary evidence that sCD40L may differ between PE and a clinically overlapping inflammatory respiratory condition, hospitalization-requiring CAP. The magnitude of effect observed in this cohort (AUC 0.951) exceeded that of D-dimer in this selected comparison. However, this should not be interpreted as evidence that sCD40L is superior to D-dimer for routine PE diagnosis. D-dimer is primarily used as a rule-out biomarker in patients with appropriate clinical probability, whereas D-dimer elevations may occur in infection, inflammation, cancer, aging, pregnancy, and other clinical states that reduce specificity [37,38,42,43]. Within the present selected hospitalized PE-versus-CAP comparison, the limited discriminatory signal of D-dimer likely reflects overlapping coagulation activation in both conditions rather than failure of D-dimer in its established clinical role. Contemporary data specifically evaluating sCD40L for differentiating established acute PE from hospitalization-requiring CAP remain scarce, supporting the exploratory nature of the present analysis.

### 4.6. Limitations

Key limitations include the single-center design, modest sample size, retrospective data structure, and selected two-gate case–control design. The study compared patients with established CTPA-confirmed PE and established hospitalization-requiring CAP rather than an unselected population presenting with acute dyspnea or suspected PE. Therefore, the apparent AUC, Youden-derived cut-off, sensitivity, specificity, PPV, and NPV may overestimate real-world diagnostic performance because spectrum bias, disease prevalence, competing diagnoses, and clinical uncertainty differ substantially in routine emergency or inpatient diagnostic pathways. Because serum samples and clinical data were analyzed retrospectively, temporal relationships between sampling, treatment initiation, symptom onset, and biomarker kinetics could not be standardized as they would be in a prospective diagnostic accuracy study. Restricting controls to hospitalization-requiring CAP may also limit generalizability to broader comparator populations. In addition, although CAP controls were selected through a chronological feasibility sampling approach, only a chronological feasibility sample of the first 40 hospitalized CAP patients meeting the clinical screening criteria was reviewed from a larger CAP pool; therefore, residual selection bias related to comparator sampling cannot be excluded. Because the same dataset was used for exploratory cut-off derivation and apparent performance estimation, the observed threshold-based performance may be optimistic. Although CTPA was performed in 29 of 34 CAP patients (85.3%), five patients with acute kidney injury could not undergo contrast-enhanced imaging. V/Q scintigraphy was not performed because it was not available in a clinically relevant timeframe for some patients and was not pursued in others because the treating team considered the clinical pre-test probability of PE to be low. Lower-extremity Doppler ultrasonography and one-year institutional follow-up did not identify venous thromboembolism in these patients; however, occult PE, particularly small or subsegmental PE, cannot be completely excluded. This introduces potential verification bias and represents an additional limitation of the selected retrospective design. The extent-stratified analyses were exploratory and underpowered, and the observed subgroup findings should be considered hypothesis-generating rather than confirmatory.

Antithrombotic medication exposure. To examine a clinically relevant potential confounder, the available dataset was reviewed for recorded antiplatelet therapy and rivaroxaban use at admission. Such exposure was present in five patients and was addressed by a medication-exclusion sensitivity analysis. However, the available retrospective fields did not provide comprehensive ascertainment of all antiplatelet or anticoagulant agents, medication adherence, prior discontinuation intervals, or treatment exposure before referral. This limitation is relevant because platelet-modifying therapies can influence platelet-derived sCD40L release and circulating sCD40L concentrations [44,45]. Consequently, residual medication-related confounding remains possible, and the exploratory cut-off should not be considered clinically actionable.

Because sCD40L concentrations are sensitive to sample matrix and pre-analytical processing [40], and because the manufacturer ELISA protocol includes a fixed 1:5 sample dilution, the back-calculated serum-equivalent concentrations are subject to assay-specific calibration; absolute values and thresholds may vary across assays, laboratories, sample matrices, and pre-analytical conditions. Because serum sCD40L may partly reflect ex vivo platelet activation during clot formation, the absolute concentrations reported here should not be interpreted as directly interchangeable with platelet-poor plasma measurements. All ELISA measurements were performed in duplicate; however, independent external assay validation was not available in this retrospective exploratory study, which further supports interpreting the reported concentrations and cut-off as assay-specific and preliminary. Accordingly, the 725.5 pg/mL cut-off should be regarded as assay- and matrix-specific until external multicenter validation is performed.

## 5. Conclusions

In this retrospective selected two-group comparison, on-admission serum sCD40L concentrations were higher in established acute PE than in hospitalization-requiring CAP. The observed ROC findings indicate an apparent within-sample discriminatory signal only and should be considered hypothesis-generating. Nevertheless, the retrospective selected design, modest sample size, incomplete capture of potential medication and cardiovascular confounders, incomplete CTPA verification in a small CAP subset, absence of external validation, and ELISA-based batch measurement preclude definitive clinical conclusions. sCD40L should not be considered a replacement for D-dimer, clinical probability assessment, or imaging-based PE diagnosis. Prospective multicenter validation in unselected suspected-PE populations with comprehensive medication ascertainment, systematic PE and CAP extent stratification, standardized sampling before treatment initiation, and standardized rapid assay platforms would be required before any clinical implementation.

## Figures and Tables

**Table 1:** Baseline clinical characteristics and comorbidities of PE and hospitalization-requiring CAP cohorts.

Parameter	Pulmonary Embolism (*n* = 48)	Hospitalization-Requiring CAP (*n* = 34)	*p*
Sex (male/female), *n*	22/26	20/14	0.251
Age, years (mean ± SD)	68.83 ± 17.42	71.91 ± 14.64	0.389
Diabetes mellitus, *n* (%)	0 (0.0)	5 (14.7)	0.023 *
Hypertension, *n* (%)	5 (10.4)	5 (14.7)	0.575
Chronic obstructive pulmonary disease, *n* (%)	10 (20.8)	17 (50.0)	0.008 *
Malignancy, *n* (%)	8 (16.7)	5 (14.7)	1.000
Chronic renal failure, *n* (%)	3 (6.3)	0 (0.0)	0.263
Atrial fibrillation, *n* (%)	0 (0.0)	4 (11.8)	0.027 *
Documented antiplatelet therapy at admission, *n* (%)	1 (2.1)	0 (0.0)	1.000
Documented rivaroxaban use at admission, *n* (%)	0 (0.0)	4 (11.8)	0.027 *
Documented antithrombotic exposure, any, *n* (%)	1 (2.1)	4 (11.8)	0.155

Values are *n* (%) unless otherwise indicated. For sparse categorical comparisons, Fisher’s exact test was used. Documented antithrombotic exposure denotes available-record evidence of antiplatelet therapy or rivaroxaban use at admission. SD, standard deviation; PE, pulmonary embolism; CAP, community-acquired pneumonia; COPD, chronic obstructive pulmonary disease. * *p* < 0.05.

**Table 2:** PE severity stratification within the PE cohort (*n* = 48).

Variable	Value	Notes
Revised Geneva score, median (IQR)	10 (7–12)	—
Geneva: Low probability (0–3), *n* (%)	2 (4.2%)	—
Geneva: Intermediate (4–10), *n* (%)	23 (47.9%)	—
Geneva: High (≥11), *n* (%)	23 (47.9%)	—
PESI class I, *n* (%)	10 (20.8%)	—
PESI class II, *n* (%)	3 (6.2%)	—
PESI class III, *n* (%)	2 (4.2%)	—
PESI class IV, *n* (%)	13 (27.1%)	—
PESI class V, *n* (%)	20 (41.7%)	—
Low-risk PE, *n* (%)	6 (12.5%)	—
Intermediate-risk PE, *n* (%)	31 (64.6%)	—
High-risk PE, *n* (%)	11 (22.9%)	—

PESI, Pulmonary Embolism Severity Index; IQR, interquartile range.

**Table 3:** Comparison of laboratory parameters between PE and hospitalization-requiring CAP patients.

Variable	PE Median (Q1–Q3)	CAP Median (Q1–Q3)	*p*
sCD40L (serum-equivalent, pg/mL)	821.3 (770.8–936.0)	629.0 (548.5–707.6)	<0.001 *
D-dimer (ng/mL FEU)	1671 (686–3766)	1972 (1077–3397)	0.505
Fibrinogen (mg/dL)	460.0 (319.5–547.0)	452.0 (299.0–572.3)	0.792
Neutrophil (%)	61.0 (52.0–75.3)	77.0 (69.0–87.0)	0.002 *
MCV (fL)	86.5 (82.0–91.0)	89.0 (84.0–92.0)	0.351
P-LCR (%)	27.5 (20.0–33.0)	28.0 (22.3–33.0)	0.415
Lymphocyte (%)	20.0 (4.0–33.5)	13.0 (9.0–22.8)	0.384
MCHC (g/dL)	32.0 (31.0–33.0)	32.0 (31.0–32.0)	0.047 *
Troponin I (ng/L)	17.0 (5.0–330.5)	25.0 (10.3–97.0)	0.697
CRP (mg/L)	61.0 (24.0–174.5)	41.0 (19.3–108.0)	0.174
PCT (ng/mL)	0.15 (0.01–0.25)	0.10 (0.01–0.89)	0.767
WBC (×10^3^/µL)	9.0 (7.0–14.0)	9.0 (7.3–12.0)	0.809
Platelet count (×10^3^/µL)	229.5 (199.8–304.8)	231.0 (170.5–317.8)	0.534
MPV (fL)	9.95 (9.47–10.45)	10.30 (9.60–11.10)	0.079
PDW (%)	11.00 (9.70–12.38)	11.25 (10.07–13.43)	0.106
NLR	3.55 (1.66–22.25)	6.08 (3.89–12.43)	0.271
PLR	157.7 (65.0–858.3)	219.2 (97.4–410.2)	0.847
SII	966.5 (395.0–4525.5)	1551.6 (830.1–3046.9)	0.571
Albumin (g/L)	39.5 (35.0–44.0)	34.5 (31.3–40.0)	0.005 *
LDH (U/L)	293.5 (243.5–352.0)	267.0 (232.8–362.3)	0.661

Values are presented as median (Q1–Q3); *p* values from Mann–Whitney U test. Available-case analysis was used for laboratory comparisons. PE, pulmonary embolism; CAP, community-acquired pneumonia; sCD40L, soluble CD40 ligand; PCT, procalcitonin; CRP, C-reactive protein; WBC, white blood cell count; MCV, mean corpuscular volume; MCHC, mean corpuscular hemoglobin concentration; P-LCR, platelet large cell ratio; MPV, mean platelet volume; PDW, platelet distribution width; NLR, neutrophil-to-lymphocyte ratio; PLR, platelet-to-lymphocyte ratio; SII, systemic immune-inflammation index; LDH, lactate dehydrogenase; FEU, fibrinogen-equivalent units. * *p* < 0.05.

**Table 4:** Exploratory within-sample classification at the Youden-derived sCD40L threshold of 725.5 pg/mL.

Sensitivity	93.8% (45/48; 95% CI 83.2–97.9)	PPV	90.0% (45/50; 95% CI 78.6–95.7)
Specificity	85.3% (29/34; 95% CI 69.9–93.6)	NPV	90.6% (29/32; 95% CI 75.8–96.8)
LR+	6.38 (95% CI 2.83–14.37)	LR−	0.07 (95% CI 0.02–0.22)

PPV, positive predictive value; NPV, negative predictive value; LR, likelihood ratio; CI, confidence interval. Classification counts: PE test-positive/test-negative = 45/3; CAP test-positive/test-negative = 5/29. Confidence intervals for proportions are Wilson 95% CIs; likelihood-ratio CIs are approximate log-method 95% CIs. PPV and NPV are within-sample descriptive values only because of the selected case–control design.

**Table 5:** Documented antithrombotic exposure and atrial fibrillation at admission by diagnostic group.

Variable	PE (*n* = 48)	CAP (*n* = 34)	*p* Value
Documented antiplatelet therapy, *n* (%)	1 (2.1)	0 (0.0)	1.000
Documented rivaroxaban use, *n* (%)	0 (0.0)	4 (11.8)	0.027
Documented antithrombotic exposure, any, *n* (%)	1 (2.1)	4 (11.8)	0.155
Atrial fibrillation, *n* (%)	0 (0.0)	4 (11.8)	0.027

**Table 6:** Firth-penalized logistic regression in the medication-exclusion sensitivity cohort (*n* = 77; 47 acute PE and 30 hospitalization-requiring CAP patients).

Predictor	Adjusted OR	95% CI	*p* Value
sCD40L (per 50 pg/mL increase)	3.39	2.00–7.71	<0.001
Platelet count (per 50 × 10^3^/µL increase)	0.91	0.59–1.39	0.677
COPD (yes vs. no)	0.74	0.13–4.19	0.720

**Table 7:** Exploratory within-sample ROC summaries of selected biomarkers in the PE-versus-CAP comparison.

Parameter	Cut-Off (Youden)	Sens %	Spec %	PPV %	NPV %	AUC (95% CI)	LR+	LR−
sCD40L (pg/mL, serum-equivalent)	725.5 pg/mL	93.8	85.3	90.0	90.6	0.951 (0.905–0.997)	6.38	0.07
D-dimer (ng/mL FEU)	4768 ng/mL FEU	25.6	85.3	68.8	47.5	0.483 (0.355–0.610)	1.74	0.87
PCT (ng/mL)	0.13 ng/mL	54.2	56.7	66.7	43.6	0.480 (0.347–0.613)	1.25	0.81
Neutrophil %	68%	64.6	75.8	79.5	59.5	0.703 (0.584–0.821)	2.67	0.47

Note: These estimates are descriptive within-sample summaries from a selected two-gate cohort and should not be interpreted as evidence of clinical diagnostic superiority or as clinically actionable thresholds.

**Table 8:** Comparison of laboratory parameters among PE patients with and without right ventricular dysfunction (RVD).

Parameter	Without RVD	With RVD	*p*
sCD40L (pg/mL, serum-equivalent)	804.0 (748.6–898.5)	831.8 (779.6–957.6)	0.198
D-dimer (ng/mL FEU)	1806 (492–4685)	1535 (910–2501)	0.990
PCT (ng/mL)	0.18 (0.01–0.37)	0.14 (0.02–0.24)	0.889
Troponin I (ng/L)	10.0 (4.5–25.0)	52.5 (5.0–369.5)	0.202
Lymphocyte %	15.0 (4.5–25.8)	23.0 (4.0–37.8)	0.412
Neutrophil %	63.0 (59.2–75.0)	60.5 (52.0–75.2)	0.572
WBC (×10^3^/µL)	7.0 (7.0–11.0)	9.5 (8.0–14.0)	0.121
Fibrinogen (mg/dL)	480.0 (302.0–551.0)	434.0 (325.0–529.0)	0.991

Values presented as median (Q1–Q3). *p* values from Mann–Whitney U test. RVD, right ventricular dysfunction. Group sizes: Without RVD *n* = 18 and With RVD *n* = 30; valid *n* varies by parameter due to missing data.

## Data Availability

The de-identified datasets analyzed during the current study are available from the corresponding author on reasonable request, subject to approval by the local ethics committee.

## Supplementary Materials

The following supporting information can be downloaded at: https://www.mdpi.com/article/10.3390/diagnostics16121877/s1, Supplementary Figure S1: Screening flow diagram and derivation of the analytic cohort; Supplementary Table S1: Exploratory alternative sCD40L thresholds in the full cohort; Supplementary Table S2: Performance of the original exploratory sCD40L threshold after exclusion of patients with recorded antithrombotic exposure at admission; Supplementary Table S3: Exploratory disease-extent stratification and sCD40L levels; Supplementary Table S4: Exploratory Spearman correlations between sCD40L and D-dimer; STARD 2015 Checklist; STROBE Checklist.

## Abbreviations

AUC: area under the curve; BMI: body mass index; BUN: blood urea nitrogen; CAP: community-acquired pneumonia; CI: confidence interval; CKD-EPI: Chronic Kidney Disease Epidemiology Collaboration; COPD: chronic obstructive pulmonary disease; CRP: C-reactive protein; CTEPH: chronic thromboembolic pulmonary hypertension; CTPA: computed tomography pulmonary angiography; CURB-65: confusion, urea, respiratory rate, blood pressure, and age ≥ 65 years; CV: coefficient of variation; ELISA: enzyme-linked immunosorbent assay; ESC: European Society of Cardiology; FEU: fibrinogen-equivalent units; IQR: interquartile range; LDH: lactate dehydrogenase; LR: likelihood ratio; MCHC: mean corpuscular hemoglobin concentration; MCV: mean corpuscular volume; MPV: mean platelet volume; NLR: neutrophil-to-lymphocyte ratio; OR: odds ratio; PCT: procalcitonin; PDW: platelet distribution width; PE: pulmonary embolism; PESI: Pulmonary Embolism Severity Index; PLR: platelet-to-lymphocyte ratio; PPV: positive predictive value; NPV: negative predictive value; NT-proBNP: N-terminal pro-B-type natriuretic peptide; ROC: receiver operating characteristic; RVD: right ventricular dysfunction; SD: standard deviation; SII: systemic immune-inflammation index; sCD40L: soluble CD40 ligand; STARD: Standards for Reporting of Diagnostic Accuracy Studies; STROBE: Strengthening the Reporting of Observational Studies in Epidemiology; TAPSE: tricuspid annular plane systolic excursion; TNF: tumor necrosis factor; TTE: transthoracic echocardiography; WBC: white blood cell count.
