# Supplementary material for: Exploratory Diagnostic Performance of On-Admission Soluble CD40 Ligand for Distinguishing Acute Pulmonary Embolism from Hospitalization-Requiring Community-Acquired Pneumonia: A Single-Center Observational Study
Source: Diagnostics (Basel). 2026 Jun 16;16(12):1877. doi: 10.3390/diagnostics16121877 (PMC13298798; doi:10.3390/diagnostics16121877)
Supplement: Supplementary file 1 [file diagnostics-16-01877-s001.zip › STARD_2015_completed_checklist_sCD40L_PE_CAP_final_updated.pdf]

STARD 2015 Checklist

Exploratory Diagnostic Performance of On-Admission Soluble CD40 Ligand for Distinguishing Acute Pulmonary Embolism from Hospitalization-Requiring Community-Acquired Pneumonia: A Single-Center Observational Study

Completed checklist for revised submission

Checklist completed for secondary exploratory ROC-based analyses within a selected PE-versus-CAP comparative cohort. The manuscript explicitly states that these analyses are within-sample and not intended to validate a clinically actionable diagnostic test.

Item	Topic	STARD 2015 item	Reported?	Manuscript location / Comments
1	Identification as diagnostic accuracy study	Identify the article as a study of diagnostic accuracy in at least one of the title, abstract, or keywords.	Partial / Not fully applicable	The title retains diagnostic-performance wording, but the Abstract, Methods, Results, Discussion, and Conclusions explicitly clarify that the ROC analyses are secondary exploratory within-sample summaries in a selected comparative cohort, not validation of diagnostic accuracy in an unselected suspected-PE population.
2	Structured summary	Provide a structured summary including design, methods, results, and conclusions.	Yes	Abstract contains Background/Objectives, Methods, Results, and Conclusions with cautious interpretation.
3	Scientific and clinical background	Describe scientific and clinical background, including intended use and clinical role of the index test.	Yes	Introduction and Clinical Implications: PE-CAP overlap, sCD40L biology, and non-replacement of D-dimer/CTPA.
4	Study objectives and hypotheses	State study objectives and hypotheses.	Yes	Introduction final paragraph: primary level comparison; secondary exploratory ROC and sensitivity analyses.
5	Study design	Specify whether data collection was planned before index/reference tests (prospective) or after (retrospective).	Yes	Methods 2.2: single-center retrospective exploratory comparative biomarker study.
6	Eligibility criteria	State eligibility criteria.	Yes	Methods 2.2-2.6: source population, diagnostic definitions, exclusions, comorbidities.
7	Identifying eligible participants	State the basis for identifying potentially eligible participants.	Yes	Methods 2.2: 50 hospitalized acute PE and 144 CAP patients were identified in the Chest Diseases Clinic; all available PE cases and a chronological feasibility sample of the first 40 CAP admissions meeting clinical screening criteria were reviewed.
8	Setting and dates	Describe setting, location, and dates of participant identification and data collection.	Yes	Methods 2.2: Erzurum Training and Research Hospital; December 2023-December 2024.
9	Participant sampling	Specify whether participants formed a consecutive, random, or convenience series.	Yes	Methods 2.2: all available PE cases and a chronological feasibility sample of the first 40 CAP admissions meeting clinical screening criteria were reviewed from the source population.
10a	Index test details	Describe the index test in sufficient detail to allow replication.	Yes	Methods 2.9: serum sCD40L ELISA kit, catalog no., assay range, CV, dilution, duplicate measurement, sample handling.
10b	Index test positivity/cutoffs	State how index test results were defined and rationale for thresholds.	Yes	Methods 2.10/Results 3.5: Youden-derived threshold reported descriptively only; not clinically actionable.
11	Reference standard details/rationale	Describe reference standard and rationale.	Yes	Methods 2.3/2.5: CTPA-confirmed acute PE; CAP clinical/radiological definition; CTPA/Doppler/follow-up handling in CAP.
12a	Clinical information available	State whether clinical information and reference standard results were available to index-test performers/readers.	Partial	sCD40L was measured in stored serum by batch ELISA; clinical diagnostic groups were defined from records. Blinding of ELISA operators is not explicitly stated.
12b	Clinical information available to reference standard assessors	State whether index test results were available to reference standard assessors.	Yes	Reference standard diagnosis was based on clinical imaging/records before research sCD40L batch measurement; sCD40L was not routine clinical testing.
13a	Methods for estimating accuracy	Describe methods for estimating or comparing diagnostic accuracy.	Yes	Methods 2.10: ROC/AUC, 95% CI, Youden threshold, sensitivity/specificity/likelihood ratios, bootstrap CI in sensitivity cohort.
13b	Handling indeterminate/missing results	Describe handling of indeterminate index/reference standard results.	Partial	No indeterminate sCD40L described; CTPA incomplete in five CAP patients handled via Doppler/follow-up and discussed as verification bias.

14	Adverse events	Describe methods for recording adverse events from index test or reference standard.	N/A	Retrospective stored-serum ELISA; no study-related adverse events expected.
15	Sample size	Describe how sample size was determined.	Yes	Methods 2.10: no formal a priori calculation; exploratory retrospective design; prespecified eligible screened patients included.
16	Participant flow diagram	Describe participant flow using a diagram.	Yes	Supplementary Figure S1 prepared: 50 PE/144 CAP source population, screened/excluded/included counts.
17	Dates	Report dates defining recruitment/data collection.	Yes	Methods 2.2: December 2023-December 2024.
18	Participant characteristics	Report demographic and clinical characteristics.	Yes	Results 3.1 and Table 1.
19	Distribution of disease severity/alternative diagnoses	Report distribution of severity and alternative diagnoses.	Yes	Results 3.2 and 3.3: Geneva/PESI/risk, PAOI categories, CAP extent; exclusion of overlapping PE+CAP described.
20	Time interval/interventions	Report time interval between index and reference standard and interventions between them.	Partial	Blood Sampling section: samples were obtained at or near admission; when timing was documented, sampling appeared to precede therapeutic anticoagulation or systemic antibiotic therapy, but undocumented timing variability cannot be excluded.
21	Cross-tabulation	Provide cross-tabulation of index test results by reference standard.	Yes	Table 4: PE test-positive/test-negative = 45/3; CAP = 5/29 at exploratory threshold.
22	Accuracy estimates	Report estimates of diagnostic accuracy and precision.	Yes	Results 3.5, Table 4, Table 7: AUC, sensitivity, specificity, PPV/NPV, LR with 95% CI; interpreted as exploratory within-sample estimates.
23	Indeterminate/missing/unusable results	Report number of indeterminate/missing results.	Partial	No unusable sCD40L results reported. CTPA was unavailable in five CAP patients; Doppler ultrasonography and one-year institutional follow-up are reported, and potential verification bias is acknowledged.
24	Adverse events	Report adverse events from index/reference standard.	N/A	No study-related adverse events due to retrospective stored-serum ELISA.
25	Variability of diagnostic accuracy	Report analyses of variability in diagnostic accuracy, distinguishing prespecified from exploratory.	Yes	Medication-exclusion sensitivity analysis and extent-stratified analyses are clearly described as exploratory.
26	Reproducibility	Report estimates of test reproducibility if done.	Partial	Duplicate ELISA measurements reported; formal reproducibility estimates beyond manufacturer CV were not performed.
27	Limitations	Discuss study limitations, including sources of bias and uncertainty.	Yes	Discussion/Limitations: selected two-gate design, spectrum bias, verification bias, external validation, medication and pre-analytical limitations.
28	Implications	Discuss implications for practice, including intended use and role of index test.	Yes	Clinical Implications: sCD40L not routine rapid test, not replacing D-dimer/clinical probability/CTPA; research biomarker pending validation.
29	Registration	Give registration number and registry name.	N/A	Retrospective exploratory study; no trial registration reported.
30	Protocol and funding	Indicate where the full protocol can be accessed; state funding sources and role of funders.	Partial/Yes	No publicly accessible protocol; no external funding; data available on reasonable request subject to ethics approval.

Note: Some STARD items are marked as Partial or N/A because the manuscript is primarily an observational comparative biomarker study, not a prospective diagnostic accuracy study in an unselected suspected-PE population. STARD items were addressed where relevant because secondary exploratory ROC-based analyses were performed.
