# Supplementary material for: Exploratory Diagnostic Performance of On-Admission Soluble CD40 Ligand for Distinguishing Acute Pulmonary Embolism from Hospitalization-Requiring Community-Acquired Pneumonia: A Single-Center Observational Study
Source: Diagnostics (Basel). 2026 Jun 16;16(12):1877. doi: 10.3390/diagnostics16121877 (PMC13298798; doi:10.3390/diagnostics16121877)
Supplement: Supplementary file 1 [file diagnostics-16-01877-s001.zip › STROBE_completed_checklist_sCD40L_PE_CAP_final_updated.pdf]

STROBE Statement Checklist

Exploratory Diagnostic Performance of On-Admission Soluble CD40 Ligand for Distinguishing Acute Pulmonary Embolism from Hospitalization-Requiring Community-Acquired Pneumonia: A Single-Center Observational Study

Completed checklist for revised submission

Checklist completed for a retrospective exploratory comparative biomarker study. Locations refer to sections/tables in the revised manuscript rather than fixed page numbers, because page numbering may change during journal production.

Item	Section	Recommendation	Reported?	Manuscript location / Comments
1a	Title and abstract	Indicate the study design with a commonly used term in the title or abstract.	Yes	Abstract: single-center retrospective exploratory comparative biomarker study.
1b	Title and abstract	Provide an informative and balanced summary of what was done and found.	Yes	Abstract: objectives, methods, results, and cautious conclusions; notes selected cohort and hypothesis-generating nature.
2	Background/rationale	Explain the scientific background and rationale for the investigation.	Yes	Introduction: PE-CAP overlap, sCD40L thrombo-inflammatory biology, gaps in comparative data.
3	Objectives	State specific objectives, including any prespecified hypotheses.	Yes	Introduction final paragraph: primary comparison of sCD40L levels; secondary exploratory ROC and sensitivity analyses.
4	Study design	Present key elements of study design early in the paper.	Yes	Methods 2.2: single-center retrospective exploratory comparative biomarker study; selected two-gate design noted.
5	Setting	Describe setting, locations, and relevant dates, including periods of recruitment/exposure/follow-up/data collection.	Yes	Methods 2.2 and 2.5: Erzurum Training and Research Hospital; December 2023-December 2024; one-year institutional follow-up for five CAP patients without CTPA.
6a	Participants	Give eligibility criteria and sources/methods of participant selection.	Yes	Methods 2.2-2.6: 50 PE and 144 CAP source population; all available PE cases and a chronological feasibility sample of the first 40 CAP admissions meeting clinical screening criteria were reviewed; inclusion/exclusion criteria described.
6b	Participants	For matched studies, give matching criteria and number of exposed/unexposed.	N/A	No matching design was used.
7	Variables	Clearly define all outcomes, exposures, predictors, potential confounders, and effect modifiers.	Yes	Methods 2.3-2.10: diagnostic groups, sCD40L, routine laboratory variables, medication exposure, COPD, PAOI, CAP extent, RVD.
8	Data sources/measurement	For each variable, give sources of data and measurement methods; describe comparability if more than one group.	Yes	Methods 2.5, 2.7-2.10: CTPA, PAOI/Qanadli categories, CAP extent, TTE, admission serum and ELISA, standard laboratory methods.
9	Bias	Describe efforts to address potential sources of bias.	Yes	Methods and Discussion: prespecified exclusions, medication-exclusion sensitivity analysis, COPD adjustment, verification bias and two-gate design discussed.
10	Study size	Explain how study size was arrived at.	Yes	Methods 2.10: no formal a priori sample-size calculation; all patients in the screened cohort who met retrospectively defined eligibility criteria before analysis had stored admission serum available and were included.
11	Quantitative variables	Explain how quantitative variables were handled; describe groupings.	Yes	Methods 2.5 and 2.10: PAOI categories, CAP extent categories, sCD40L per 50 pg/mL in Firth model, summary statistics.
12a	Statistical methods	Describe all statistical methods, including those used to control for confounding.	Yes	Methods 2.10: Mann-Whitney, Kruskal-Wallis, ROC, bootstrap CI, Firth-penalized logistic regression adjusted for platelet count and COPD.
12b	Statistical methods	Describe methods used to examine subgroups and interactions.	Yes	Methods 2.10 and Results 3.3/3.8: PE obstruction burden, CAP extent, PE vs extensive CAP, RVD subgroup.
12c	Statistical methods	Explain how missing data were addressed.	Yes	Methods/Results: available-case analyses; CTPA unavailable in five CAP patients, with Doppler/follow-up information provided and potential verification bias acknowledged.
12d	Statistical methods	Cohort study: explain loss to follow-up; case-control: explain matching; cross-sectional: describe sampling strategy.	Partial	Sampling strategy described as a retrospective selected two-gate comparative biomarker design rather than a classical cohort or case-control design; one-year institutional follow-up described for five CAP patients without CTPA.
12e	Statistical methods	Describe sensitivity analyses.	Yes	Methods 2.10 and Results 3.6: medication-exclusion sensitivity cohort,

				bootstrap AUC, Firth model.
13a	Participants	Report numbers of individuals at each stage.	Yes	Methods 2.2 and Supplementary Figure S1: 50 PE and 144 CAP source population; 50 PE and first 40 CAP screened; 48 PE and 34 CAP included.
13b	Participants	Give reasons for non-participation at each stage.	Yes	Methods 2.2 and Supplementary Figure S1: UTI, previous arterial thrombosis, severe valvular disease, recent CVA, extrapulmonary infection.
13c	Participants	Consider use of a flow diagram.	Yes	Supplementary Figure S1 prepared for submission.
14a	Descriptive data	Give characteristics of study participants.	Yes	Results 3.1 and Table 1.
14b	Descriptive data	Indicate number of participants with missing data for each variable of interest.	Partial	Partial: available-case analyses were used; Table 8 notes that valid n varies by parameter for RVD-related analyses.
14c	Descriptive data	Cohort study: summarize follow-up time.	N/A/Partial	Not a longitudinal cohort; one-year institutional follow-up is described only for five CAP patients without CTPA.
15	Outcome data	Report numbers of outcome events or summary measures.	Yes	Results: group counts; sCD40L medians; extent categories; ROC summaries; RVD subgroup.
16a	Main results	Give unadjusted estimates and, if applicable, confounder-adjusted estimates with precision.	Yes	Results 3.4-3.7 and Table 6: group differences, ROC/AUC, and adjusted Firth OR with 95% CI are reported; Table 5 reports documented antithrombotic exposure.
16b	Main results	Report category boundaries when continuous variables were categorized.	Yes	Methods 2.5: PAOI low <20%, intermediate 20-37.5%, high >=40%; CAP limited/extensive definitions.
16c	Main results	If relevant, translate relative risk into absolute risk.	N/A	No relative risk estimates are the focus.
17	Other analyses	Report other analyses done, e.g., subgroup and sensitivity analyses.	Yes	Results 3.3, 3.6, 3.8: extent-stratified analyses, PE vs extensive CAP, D-dimer correlation, medication-exclusion sensitivity, RVD analysis.
18	Discussion: key results	Summarize key results with reference to study objectives.	Yes	Discussion opening paragraph.
19	Discussion: limitations	Discuss limitations, considering sources of potential bias or imprecision.	Yes	Limitations: selected two-gate design, modest sample size, incomplete CTPA verification, medication confounding, pre-analytical variability, and non-standardized timing of sampling, treatment initiation, symptom onset, and biomarker kinetics.
20	Discussion: interpretation	Give a cautious overall interpretation considering objectives, limitations, multiplicity, and evidence.	Yes	Discussion and Conclusion: exploratory/hypothesis-generating; not replacing D-dimer, clinical probability, or imaging.
21	Discussion: generalisability	Discuss generalisability of the study results.	Yes	Discussion/Limitations: cannot extrapolate to unselected suspected-PE or acute dyspnea populations; prospective validation required.
22	Funding	Give source of funding and role of funders.	Yes	Funding statement: no external funding.

Note: STROBE is the primary reporting framework because the revised manuscript is observational and retrospective. STARD items are addressed separately because ROC-based analyses are secondary and exploratory.
