# Supplementary material for: Exploratory Diagnostic Performance of On-Admission Soluble CD40 Ligand for Distinguishing Acute Pulmonary Embolism from Hospitalization-Requiring Community-Acquired Pneumonia: A Single-Center Observational Study
Source: Diagnostics (Basel). 2026 Jun 16;16(12):1877. doi: 10.3390/diagnostics16121877 (PMC13298798; doi:10.3390/diagnostics16121877)
Supplement: Supplementary file 1 [file diagnostics-16-01877-s001.zip › Supplementary_Material_sCD40L_PE_CAP_final_updated.pdf]

Supplementary Materials

Exploratory Diagnostic Performance of On-Admission Soluble CD40 Ligand for Distinguishing Acute Pulmonary Embolism from Hospitalization-Requiring Community-Acquired Pneumonia: A Single-Center Observational Study

Onur Çelik, Adil Furkan Kılıç, Yunus Kuralay, Dursun Erol Afşin

This supplementary document includes the screening flow diagram, exploratory alternative sCD40L thresholds, medication-exclusion sensitivity performance, and exploratory disease-extent analyses. Completed STROBE and STARD 2015 checklists are provided as separate supplementary/non-published files in the same ZIP package.

Supplementary Figure S1. Screening flow diagram and derivation of the analytic cohort

Study period: December 2023–December 2024

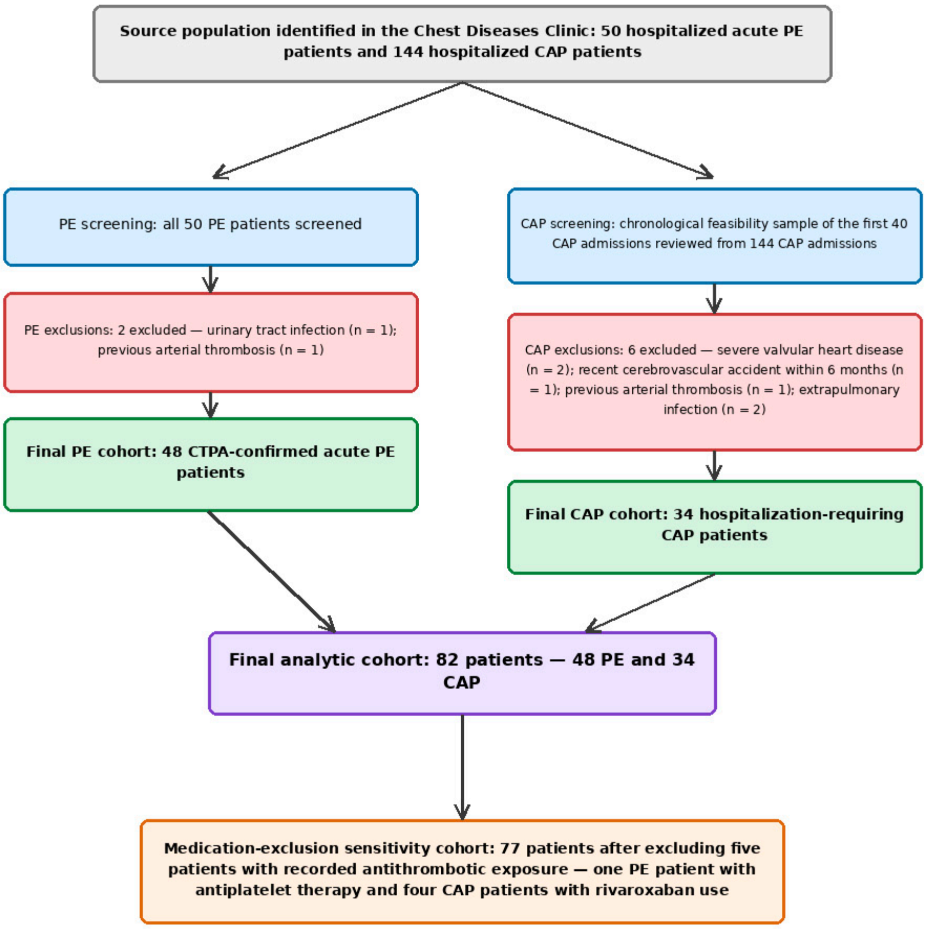

Abbreviations: CAP, community-acquired pneumonia; CTPA, computed tomography pulmonary angiography; PE, pulmonary embolism.

Supplementary Table S1. Exploratory alternative sCD40L thresholds in the full cohort

Full cohort: n = 82; 48 PE and 34 hospitalization-requiring CAP patients. sCD40L values are reported as serum-equivalent concentrations because the ELISA protocol required a fixed 1:5 sample dilution.

Threshold (pg/mL)	Exploratory intent	TP	FP	TN	FN	Sensitivity	Specificity	PPV	NPV	LR+ / LR-
621.25	High-sensitivity threshold	48	19	15	0	100.0%	44.1%	71.6%	100.0%	1.79 / 0.00
725.50	Youden-derived threshold	45	5	29	3	93.8%	85.3%	90.0%	90.6%	6.38 / 0.07
783.25	High-specificity threshold	33	1	33	15	68.8%	97.1%	97.1%	68.8%	23.37 / 0.32
800.50	100%-specificity threshold within this cohort	29	0	34	19	60.4%	100.0%	100.0%	64.2%	Not estimable / 0.40

Abbreviations: CAP, community-acquired pneumonia; FN, false negative; FP, false positive; LR, likelihood ratio; NPV, negative predictive value; PE, pulmonary embolism; PPV, positive predictive value; TN, true negative; TP, true positive. These thresholds are exploratory, assay-specific, and not clinically actionable. LR+ was not estimable at the 800.50 pg/mL threshold because no false-positive cases were observed in this selected cohort.

Supplementary Table S2. Performance of the original exploratory sCD40L threshold after exclusion of patients with recorded antithrombotic exposure at admission

Sensitivity cohort / threshold	Confusion matrix	Classification estimates	AUC (95% CI)
Medication-exclusion sensitivity cohort (n = 77; 47 PE and 30 CAP); original threshold 725.5 pg/mL	TP 44; FP 5; TN 25; FN 3	Sensitivity 93.6%; Specificity 83.3%; PPV 89.8%; NPV 89.3%; LR+ 5.62; LR− 0.08	0.945 (bootstrap 95% CI: 0.891–0.984)

Note: The medication-exclusion sensitivity cohort excluded one PE patient with documented antiplatelet therapy and four hospitalization-requiring CAP patients with documented rivaroxaban use at admission.

Supplementary Table S3. Exploratory disease-extent stratification and sCD40L levels

All analyses in this table are exploratory and underpowered and should be interpreted descriptively.

Analysis	Group	n	Median sCD40L (pg/mL)	Q1–Q3	Statistical test	p value
PE obstruction-burden categories	Low PAOI (<20%)	19	828.5	772.5–927.8	Kruskal–Wallis	0.984
PE obstruction-burden categories	Intermediate PAOI (20–37.5%)	10	807.8	773.5–915.4	Kruskal–Wallis	0.984
PE obstruction-burden categories	High PAOI (≥40%)	19	821.5	777.0–937.5	Kruskal–Wallis	0.984
CAP radiological extent	Limited CAP	15	548.5	495.3–660.5	Mann–Whitney U	0.012
CAP radiological extent	Extensive CAP	19	640.0	622.0–721.5	Mann–Whitney U	0.012
Sensitivity comparison	Full PE cohort	48	821.3	770.8–936.0	Mann–Whitney U	<0.001
Sensitivity comparison	Extensive CAP subgroup	19	640.0	622.0–721.5	Mann–Whitney U	<0.001

Abbreviations: CAP, community-acquired pneumonia; PAOI, pulmonary artery obstruction index; PE, pulmonary embolism; Q1–Q3, interquartile range. Extensive CAP was defined as multilobar and/or bilateral involvement; limited CAP was defined as unilateral single-lobar involvement.

Supplementary Table S4. Exploratory Spearman correlations between sCD40L and D-dimer

Cohort	Spearman rho	p value
Overall cohort	-0.168	0.144
PE cohort	-0.248	0.109
CAP cohort	0.001	0.996

Abbreviations: CAP, community-acquired pneumonia; PE, pulmonary embolism; sCD40L, soluble CD40 ligand.
